# Multi-gene co-mutations of *BRAF* with *TERT*, *PIK3CA*, or *TP53* are powerful predictors of central lymph node metastasis in papillary thyroid carcinoma

**DOI:** 10.3389/fendo.2025.1728045

**Published:** 2026-01-27

**Authors:** Qing Yu, Han Liu

**Affiliations:** Department of Pathology, Deyang People’s Hospital, Deyang, China

**Keywords:** lymph node metastasis, multi-gene co-mutation, next-generation sequencing (NGS), papillary thyroid carcinoma, risk factors

## Abstract

**Background:**

The accurate preoperative prediction of lymph node metastasis (LNM) in papillary thyroid carcinoma (PTC) poses a significant clinical challenge. Although clinicopathological features are commonly utilized, their predictive accuracy remains limited, and the role of multi-gene co-mutations is not fully understood.

**Objective:**

This study aimed to develop and validate an integrated risk model that combines next-generation sequencing (NGS) data with clinicopathologic features for the preoperative prediction of LNM in PTC.

**Methods:**

We retrospectively analyzed 521 patients with PTC. Gene mutations were analyzed using NGS. Independent risk factors for central (CLNM) and lateral (LLNM) lymph node metastasis were identified through univariate and multivariate logistic regression analyses.

**Results:**

The *BRAF* V600E mutation was the most prevalent (82.15%). Notably, high-risk multi-gene co-mutations **—**specifically, *BRAF* V600E co-occurring with *TERT*, *PIK3CA*, and/or *TP53*)**—**were identified as the strongest independent risk factor for CLNM (odds ratio [OR] = 6.319, 95% confidence interval [CI]: 1.738–22.976, P = 0.005). Other significant risk factors included male sex, age <45 years, bilateral lesions, tumor size >1 cm, lymphovascular invasion (LVI), and extrathyroidal extension,with gross ETE demonstrating the highest ORs (> 21).

**Conclusion:**

Preoperative NGS profiling, particularly the detection of high-risk multi-gene co-mutations, provides a powerful tool for refined risk assessment. This molecularly guided strategy has the potential to inform personalized surgical planning directly, optimizing the extent of lymph node dissection to improve oncologic outcomes while minimizing unnecessary morbidity.

## 1 Introduction

Papillary thyroid carcinoma (PTC) is the most prevalent endocrine malignancy, accounting for approximately 80% of all thyroid cancer cases (1). While generally associated with a favorable prognosis, a significant clinical challenge is the high frequency of cervical lymph node metastasis (LNM) at diagnosis, particularly involving the central lymph node metastasis (CLNM) and lateral lymph node metastasis (LLNM) compartments (2). Accurate preoperative assessment of LNM status is crucial, as it directly influences the decision to perform and the extent of prophylactic lymph node dissection, thereby impacting both oncological outcomes and surgical-related morbidity (3, 4).

Currently, preoperative risk stratification primarily relies on ultrasonography and clinicopathological features such as tumor size and location (5, 6). However, the sensitivity and specificity of imaging for detecting CLNM, especially in occult metastases, remain suboptimal (7). Furthermore, while certain clinical factors are associated with higher risk, they lack the precision needed for truly individualized surgical planning.

In the era of precision medicine, molecular markers have emerged as promising tools for refining risk assessment. The *BRAF* V600E mutation, the most common genetic alteration in PTC, has been extensively studied for its association with more aggressive clinicopathological characteristics, including LNM (8, 9). Nevertheless, the predictive power of *BRAF* V600E as a standalone marker is often debated and appears insufficiently robust to guide surgical decisions alone (10, 11). This limitation highlights the complexity of tumorigenesis and suggests that the aggressive behavior of PTC may be driven not by a single gene, but by the synergistic effects of multiple genetic alterations. For instance, co-occurring mutations in genes such as *TERT* (promoting cellular immortality), *PIK3CA* (enhancing invasion), and *TP53* (disabling apoptosis) are hypothesized to collaborate with *BRAF*, leading to a more metastatic phenotype (12, 13). Previous studies, such as those by Vuong et al. (14), have begun to highlight the prognostic superiority of dual mutations (e.g., *BRAF*+*TERT*) over single markers.

However, while recent studies have significantly advanced our understanding of specific dual mutations, such as the prognostic impact of concurrent *BRAF* V600E and *TERT* promoter mutations (14) and the role of *BRAF*/*TP53* co-alterations (15), the synergistic effect of broader, predefined multi-gene constellations (specifically *BRAF* co-mutated with *TERT*, *PIK3CA*, and/or *TP53*), their differential impact on LNM patterns (CLNM vs. LLNM), and crucially, their quantifiable value when integrated into a preoperative clinical prediction model, remain underexplored.

Therefore, to address this gap, we conducted a retrospective study utilizing high-throughput next-generation sequencing (NGS) in a large, well-characterized Chinese cohort of 521 PTC patients. We aimed to systematically profile the mutation landscape, investigate the synergistic effects of these specific multi-gene co-mutations, and ultimately integrate these molecular signatures with conventional clinicopathological parameters to construct and validate a quantitative, preoperative prediction model (nomogram). The goal is to develop a clinically translatable, integrated (molecular-clinicopathological) tool that guides individualized surgical decision-making regarding the necessity and extent of lymph node dissection (10, 11).

## 2 Materials and methods

### 2.1 Materials

This was a single-center, retrospective, exploratory study. It aimed to investigate the feasibility of integrating multi-gene co-mutation patterns into preoperative risk assessment. It included all consecutive patients diagnosed with PTC by postoperative pathology at Deyang People’s Hospital between June 2022 and January 2025, who had undergone next-generation sequencing (NGS) testing (n=521).

The inclusion and exclusion criteria were as follows:

#### 2.1.1 Inclusion criteria

(1) Postoperative histopathological confirmation of PTC; (2) Availability of formalin-fixed paraffin-embedded (FFPE) tumor tissue from the surgical specimen for molecular testing; (3) Completion of a standardized NGS assay.

#### 2.1.2 Exclusion criteria

(1) History of any other malignant tumor; (2) Prior treatment for thyroid cancer (e.g., radiotherapy, chemotherapy, or targeted therapy); (3) Incomplete clinicopathological or molecular data.

NGS testing was performed postoperatively on FFPE tissue sections from the primary tumor as part of the standard diagnostic workflow. Clinicopathological features and NGS results were retrospectively analyzed.

The pathological diagnosis of PTC and evaluation of all histopathological features were independently reviewed and confirmed by two board-certified pathologists according to the World Health Organization (WHO) classification criteria. Surgical procedures included thyroid lobectomy + isthmusectomy/total thyroidectomy + central/lateral cervical lymph node dissection. The extent of cervical lymph node dissection was performed in accordance with the 2012 American Thyroid Association management guidelines (16).All patients were treatment-naïve at the time of surgery, with no prior history of radiotherapy, chemotherapy, or other targeted therapies before surgery. This study was approved by the Ethics Committee of Deyang People’s Hospital.

Among the 521 enrolled patients, the majority were female (72.4%) and under 45 years of age (65.5%). Most tumors were ≤1 cm in diameter (55.7%), unifocal (77.2%), and of the classical variant (95.6%). Lymphovascular invasion (LVI) was present in 8.6% of cases. The *BRAF* V600E mutation was prevalent (82.1%), while high-risk multi-gene co-mutations, defined as *BRAF* V600E co-mutated with *TERT*, *PIK3CA*, and/or *TP53*, were relatively rare (3.8%). We acknowledge that the limited sample size of this key subgroup constrains the statistical power for detailed interaction analyses.

### 2.2 Targeted sequencing and bioinformatic analysis

Targeted sequencing was performed using an in-house, laboratory-developed test (LDT) for thyroid cancer, which is based on a standardized commercial workflow. Genomic DNA was extracted from formalin-fixed, paraffin-embedded (FFPE) tumor samples using the QIAamp DNA FFPE Tissue Kit (Qiagen, Germany). Sequencing libraries were constructed using the Thyroid Cancer 88-Gene Targeted Sequencing Kit (RuiJing Biosciences, China). This panel targets the full exonic regions of 86 genes and specific intronic regions for the detection of fusions in 6 genes (88 genes in total; for the complete gene list see **Supplementary Table 1**), covering key driver genes such as *BRAF*, *TERT*, *RAS*, *TP53*, and *PIK3CA*. High-depth sequencing was conducted on an Illumina NextSeq 550 platform (Illumina, Canada) to achieve an average sequencing depth of >500×.

Bioinformatic analysis followed the vendor’s (RuiJing Biosciences) standard pipeline. Raw sequencing data underwent adapter trimming and quality filtering. Clean reads were aligned to the human reference genome (GRCh37/hg19), followed by duplicate read marking. Variant calling for single nucleotide variants and small insertions/deletions was performed following the Genome Analysis Toolkit (GATK) best practices. Identified variants were annotated against public databases (dbSNP, ClinVar, and COSMIC) and classified according to established clinical guidelines (e.g., AMP/ASCO/CAP).

Rigorous quality control was applied throughout the process. Key metrics assessed included tumor cell content (minimum 10%), DNA input quantity, library complexity, sequencing quality (e.g., Q30 score >80%), and on-target rate. Only samples passing all predefined QC thresholds were included in the downstream analysis.

### 2.3 Statistical methods

Statistical analysis was performed using SPSS 25.0 software. Categorical variables were expressed as frequencies (%). Differences between groups were assessed using univariate analysis (Chi-square or Fisher’s exact test, as appropriate). To ensure comprehensive and transparent reporting, all pre-specified clinicopathological and molecular variables were included in this univariate analysis, and their complete results are presented (in **Tables 1**, **2**, **3**), regardless of statistical significance.

**Table 1:** Baseline clinicopathological characteristics of the study population (n=521).

Characteristic	Total (n=521)	CLNM negative (n=236)	CLNM positive (n=285)	P value
Age, years				<0.001
<45	341 (65.5)	134 (39.3)	207 (60.7)	
≥45	180 (34.5)	102 (56.7)	78 (43.3)	
Sex				<0.001
Female	377 (72.4)	190 (50.4)	187 (49.6)	
Male	144 (27.6)	46 (31.9)	98 (68.1)	
Maximum Tumor Diameter, cm				<0.001
≤1	290 (55.7)	152 (52.4)	138 (47.6)	
>1	231 (44.3)	84 (36.4)	147 (63.6)	
Multifocality				0.673
Unifocal	402 (77.2)	186 (46.3)	216 (53.7)	
Multifocal	119 (22.8)	50 (42.0)	69 (58.0)	
Bilateral Lesions				0.008
No	412 (79.1)	201 (48.8)	211 (51.2)	
Yes	109 (20.9)	35 (32.1)	74 (67.9)	
Pathological Subtype				0.030
Classical Variant	498 (95.6)	222 (44.6)	276 (55.4)	
Follicular Variant	16 (3.1)	12 (75.0)	4 (25.0)	
Aggressive Variants*	7 (1.3)	2 (28.6)	5 (71.4)	
Extrathyroidal Extension				<0.001
None	109 (20.9)	68 (62.4)	41 (37.6)	
microscopic	393 (75.4)	167 (42.5)	226 (57.5)	
gross	19 (3.6)	1 (5.3)	18 (94.7)	
Lymphovascular Invasion				<0.001
Absent	476 (91.4)	230 (48.3)	246 (51.7)	
Present	45 (8.6)	6 (13.3)	39 (86.7)	
*BRAF* V600E Mutation				0.215
Absent	93 (17.9)	48 (51.6)	45 (48.4)	
Present	428 (82.1)	188 (43.9)	240 (56.1)	
High-Risk Co-mutation†				0.006
Absent	501 (96.2)	233 (46.5)	268 (53.5)	
Present	20 (3.8)	3 (15.0)	17 (85.0)	

Data are presented as n (%). CLNM, central lymph node metastasis.

*Aggressive variants include Tall Cell, Columnar Cell, Hobnail, and Sclerosing variants.

†High-risk multi-gene co-mutations were defined as *BRAF* V600E co-mutated with *TERT*, *PIK3CA*, and/or *TP53*.

**Table 2:** Univariate analysis of risk factors for lymph node metastasis [n (%)].

Clinicopathological parameter	Central lymph node metastasis		P value	Lateral cervical lymph node metastasis		P value
	Negative	Positive		Negative	Positive	
Sex						
Female	190(50.40)	187(49.60)	<0.001	294(77.98)	83(22.02)	0.042
Male	46(31.90	98(68.10)		100(69.44)	44(30.56)	
Age(years)						
<45	134(39.30)	207(60.70)	<0.001	244(71.55)	97(28.45)	0.003
≥45	102(56.70)	78(43.30)		150(83.33)	30(16.67)	
Maximum tumor diameter (cm)						
≤1	152(52.40)	138(47.60)	<0.001	254(87.59)	36(12.41)	<0.001
>1	84(36.40)	147(63.60)		140(60.61)	91(39.39)	
Number of lesions						
1	186(46.30)	216(53.70)	0.673	306(76.12)	96(23.88)	0.423
2	34(41.00)	49(59.00)		64(77.11)	19(22.89)	
≥3	16(44.40)	20(55.60)		24(66.67)	12(33.33)	
Tumor location						
Left Lobe/Left Lobe + Isthmus	91(48.90)	96(51.10)	0.008	152(81.72)	34(18.28)	<0.001
Right Lobe/Right Lobe + Isthmus	110(48.70)	116(51.30)		184(81.42)	42(18.58)	
Bilateral/Bilateral + Isthmus	35(32.10)	74(67.90)		58(53.21)	51(46.79)	
Number of gene mutations						
0	14(48.30)	15(51.70)	0.078	18(62.10)	11(37.90)	<0.001
1	214(46.40)	247(53.60)		361(78.30)	100(21.70)	
≥2	8(25.80)	23(74.20)		15(48.40)	16(51.60)	
High-risk co-mutations TERT/PIK3CA/TP53						
Present	3(15.00)	17(85.00)	0.006	11(55.00)	9(45.00)	0.054
Absent	233(46.50)	268(53.50)		383(76.40)	118(23.60)	
Pathological subtype						
Classical Variant	222(44.60)	276(55.40)	0.030	379(76.10)	119(23.90)	0.026
Follicular Variant	12(75.00)	4(25.00)		12(75.00)	4(25.00)	
Tall Cell Variant	0(0.00)	1(100.00)		0(0.00)	1(100.00)	
Columnar Cell Variant	1(50.00)	1(50.00)		2(100.00)	0(0.00)	
Hobnail Variant	1(100.00)	0(0.00)		1(100.00)	0(0.00)	
Sclerosing Variant	0(0.00)	3(100.00)		0(0.00)	3(100.00)	
Extrathyroidal extension						
None	68(62.39)	41(37.61)	<0.001	101(92.66)	8(7.34)	<0.001
microscopic	167(42.49)	226(57.51)		289(73.54)	104(26.46)	
gross	1(5.26)	18(94.74)		4(21.05)	15(78.95)	
Perineural invasion						
Absent	232(45.00)	283(55.00)	0.519	391(75.92)	124(24.08)	0.321
Present	4(66.70)	2(33.30)		3(50.00)	3(50.00)	
Lymphovascular invasion						
Absent	230(48.30)	246(51.70)	<0.001	372(78.15)	104(21.85)	<0.001
Present	6(13.30)	39(86.70)		22(48.89)	23(51.11)	

**Table 3:** Univariate analysis of central and lateral cervical LNM [n (%)].

Clinicopathological parameters	Number of lymph node metastases		P value
	1-5	>5	
Sex			
Female	155 (74.16)	54 (25.84)	0.131
Male	70 (66.04)	36 (33.96)	
Age (years)			
<45	156 (68.72)	71 (31.28)	0.088
≥45	69 (78.41)	19 (21.59)	
Maximum tumor diameter (cm)			
≤1	128 (87.07)	19 (12.93)	<0.001
>1	97 (57.74)	71 (42.26)	
Number of lesions			
1	168 (70.89)	69 (29.11)	0.361
2	42 (77.78)	12 (22.22)	
≥3	15 (62.50)	9 (37.50)	
Tumor location			
Left Lobe/Left Lobe + Isthmus	83 (80.58)	20 (19.42)	<0.001
Right Lobe/Right Lobe + Isthmus	97 (74.62)	33 (25.38)	
Bilateral/Bilateral + Isthmus	45 (54.88)	37 (45.12)	
Number of gene mutations			
0	12 (70.59)	5 (29.41)	0.518
1	197 (72.43)	75 (27.57)	
≥2	16 (61.54)	10 (38.46)	
High-risk multi-gene co-mutations			
Present	13 (72.22)	5 (27.78)	0.939
Absent	212 (71.38)	85 (28.62)	
Pathological subtype			
Classical Variant	220 (72.37)	84 (27.63)	0.006
Follicular Variant	5 (83.33)	1 (16.67)	
Tall Cell Variant	0 (0.00)	1 (100.00)	
Columnar Cell Variant	0 (0.00)	1 (100.00)	
Hobnail Variant	0 (0.00)	0 (0.00)	
Sclerosing Variant	0 (0.00)	3 (100.00)	
Extrathyroidal extension			
None	38 (86.36)	6 (13.64)	<0.001
microscopic	183 (72.33)	70 (27.67)	
gross	4 (22.22)	14 (77.78)	
Lymphovascular invasion			
Absent	202 (73.99)	71 (26.01)	0.010
Present	23 (54.76)	19 (45.24)	
Perineural invasion			
Absent	224 (71.79)	88 (28.21)	0.409
Present	1 (33.33)	2 (66.67)	

Variables with a univariate *P* value < 0.05 were considered candidate predictors for subsequent multivariate binary logistic regression, which was performed using the enter method to identify independent risk factors. Before model fitting, multicollinearity among all candidate variables was assessed using the variance inflation factor (VIF); a VIF value ≥ 5 was considered indicative of substantial collinearity, prompting the retention of only the clinically more relevant variable. Results of the logistic regression are presented as odds ratios (ORs) with 95% confidence intervals (CIs).

The discriminative ability of the final multivariate model was evaluated using the concordance index (C-index), equivalent to the area under the receiver operating characteristic(ROC)curve. The 95% confidence interval for the C-index was calculated using the DeLong method. Model calibration was assessed by comparing predicted probabilities with observed outcomes. Internal validation and overfitting correction were performed using bootstrap resampling with 1000 repetitions, as detailed in the Results section. A two-sided *P* value < 0.05 was considered statistically significant.

## 3 Results

### 3.1 Baseline characteristics of the study population

This study included 521 patients with papillary thyroid carcinoma. Their baseline clinicopathological characteristics and the associations with central lymph node metastasis (CLNM) are summarized in **Table 1**. In univariable analysis, younger age (<45 years), male sex, larger tumor diameter (>1 cm), bilateral lesions, specific pathological subtypes, extrathyroidal extension (ETE), lymphovascular invasion (LVI), and high-risk co-mutations were all significantly associated with a higher prevalence of CLNM (all *P* < 0.05). In contrast, multifocality and the BRAF V600E mutation alone showed no significant association with CLNM status.

### 3.2 Distribution of gene mutations

Mutations were detected in 94.43% (492/521) of the specimens. Single point mutations or gene fusion mutations were detected in 88.48% (461/521), while multi-gene co-mutations (≥2) were detected in 5.95% (31/521). Among these, 3.84% (20/521) were BRAF combined with TERT/PIK3CA/TP53. No mutations were detected in 5.57% (29/521) of the specimens. The detailed spectrum of gene mutations is presented in **Table 4**.

**Table 4:** Summary of gene mutation types in PTC patients detected by high-throughput sequencing.

Gene(s)	n
**Single Gene**	**435**
*BRAF* V600E	428
*TERT*	1
*HRAS*	2
*NRAS*	1
*KRAS*	2
*TP53*	1
**Multi-Gene Combinations**	**31**
*BRAF*V600E+ *TP53*+*PIK3CA*+*TERT*	1
*BRAF*V600E+*PIK3CA*	7
*BRAF* V600E+*TERT*	6
*BRAF* V600E+ *BRCA2*+ *TERT*	1
*BRAF* V600E+ *TP53*	5
*BRAF* V600E+ *MLH*	1
*BRAF* V600E+ *AKT1*	2
*BRAF* V600E+ *BRCA1*	1
*BRAF* V600E+ *NRAS*	1
*BRAF* V600E+ *HRAS*	1
*BRAF* V600E+ *KRAS*	1
*BRAF* V600E+ *POLE*	1
*BRAF* V600E+ *AXIN1*	1
*BRAF* V600E+ *PDGFRA*	1
*BRAF* V600E+ *ATM*	1
**Gene Fusions**	**26**
*RET*	20
*NTRK1*	2
*NTRK3*	2
*ALK*	2
**Wild-Type**	**29**

Note: Bold values indicate the total number of cases for each mutation category.

### 3.3 Risk factors for CLNM/LLNM

#### 3.3.1 Univariate analysis of risk factors for CLNM

CLNM was found in 54.70% (285/521) of patients. The metastasis rate was significantly higher in males, patients aged <45 years, tumor size >1 cm, bilateral lesions, high-risk co-mutations, tall cell/sclerosing subtypes, extrathyroidal extension, and Lymphovascular Invasion (LVI) (**Table 2**).

#### 3.3.2 Univariate analysis of risk factors for LLNM

LLNM was found in 24.38% (127/521) of patients. The metastasis rate was significantly higher in males, patients aged < 45 years, tumor size >1 cm, multi-gene mutations, bilateral lesions, tall cell/sclerosing subtypes, extrathyroidal extension, and Lymphovascular Invasion (LVI) (**Table 2**).

#### 3.3.3 Multivariate analysis of risk factors for CLNM/LLNM

Variables with P < 0.05 in the univariate analysis were included in the multivariate logistic regression model. Independent risk factors for CLNM (**Table 5**) included male sex, age <45 years, bilateral lesions, lymphovascular invasion (LVI), and extrathyroidal extension. High-risk multi-gene co-mutations (*BRAF* with *TERT*, *PIK3CA*, and/or *TP53*) were the strongest independent predictor (OR = 6.319, 95% CI: 1.738–22.976, P = 0.005). Independent risk factors for LLNM were: age <45 years, bilateral lesions, tumor size >1 cm, Lymphovascular Invasion (LVI), and extrathyroidal extension (**Table 6**). Multicollinearity diagnostics indicated that all variance inflation factors (VIFs) for the independent variables in the final models were close to 1 (range: 1.002–1.008), confirming the absence of substantial multicollinearity.

**Table 5:** Multivariate analysis of CLNM.

Variable	B	SE	Wals	P value	OR	95% CI
Sex (Female)	0.835	0.221	14.257	<0.001	2.305	1.494-3.557
Age (<45 years)	0.657	0.205	10.266	0.001	1.928	1.290-2.881
Location (Bilateral)	0.328	0.133	6.073	0.014	1.388	1.069-1.801
High-risk co-mutations (Yes vs No)	1.844	0.659	7.834	0.005	6.319	1.738-22.976
Lymphovascular Invasion	1.659	0.490	11.466	0.001	5.253	2.011-13.723
Extrathyroidal extension						
microscopic vs. none	0.788	0.248	10.073	0.002	2.200	1.352-3.580
gross vs. none	3.078	1.092	7.941	0.005	21.711	2.553-184.642

**Table 6:** Multivariate analysis of LLNM.

Variable	B	SE	Wals	P value	OR	95% CI
Age (<45 years)	0.562	0.266	4.473	0.034	1.753	1.042-2.950
Location (Bilateral)	0.757	0.158	22.847	<0.001	2.132	1.563-2.908
Size (>1 cm)	1.179	0.246	22.954	<0.001	3.250	2.007-5.264
Lymphovascular Invasion	0.947	0.383	6.108	0.013	2.579	1.236-5.506
Extrathyroidal extension						
microscopic vs. none	1.212	0.412	8.666	0.003	3.361	1.500-7.532
gross vs. none	3.091	0.751	16.920	<0.001	21.994	5.043-95.913

#### 3.3.4 Development of the predictive nomogram

**Figure 1:**
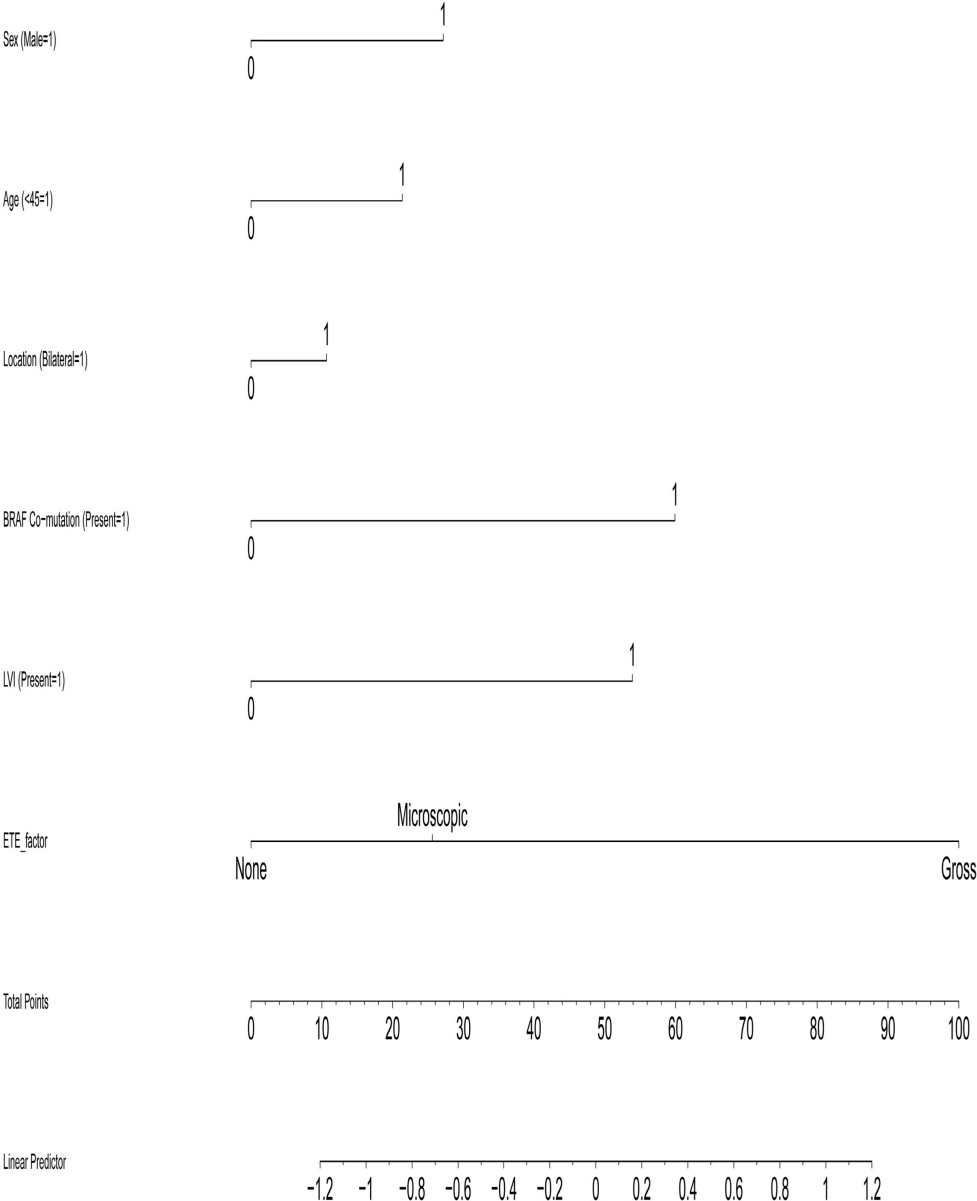
Nomogram for preoperative prediction of central lymph node metastasis (CLNM) in papillary thyroid carcinoma. To use the nomogram, for each variable, locate the patient’s value on the corresponding axis and draw a line upward to the ‘Points’ axis to determine the score. Sum all points, locate the total on the ‘Total Points’ axis, and draw a line downward to the ‘Risk of CLNM’ axis to obtain the individualized probability.

Based on the multivariate logistic regression analysis, we developed an integrated molecular-clinicopathological prediction nomogram to quantify the individual probability of CLNM (**Figure 1**). This tool, which we hereafter refer to as “the nomogram,” integrates the six identified independent predictors: sex (female as reference), age (≥45 years as reference), bilateral lesions, high-risk multi-gene co-mutations, lymphovascular invasion (LVI), and extrathyroidal extension (ETE).

**Figure 2:**
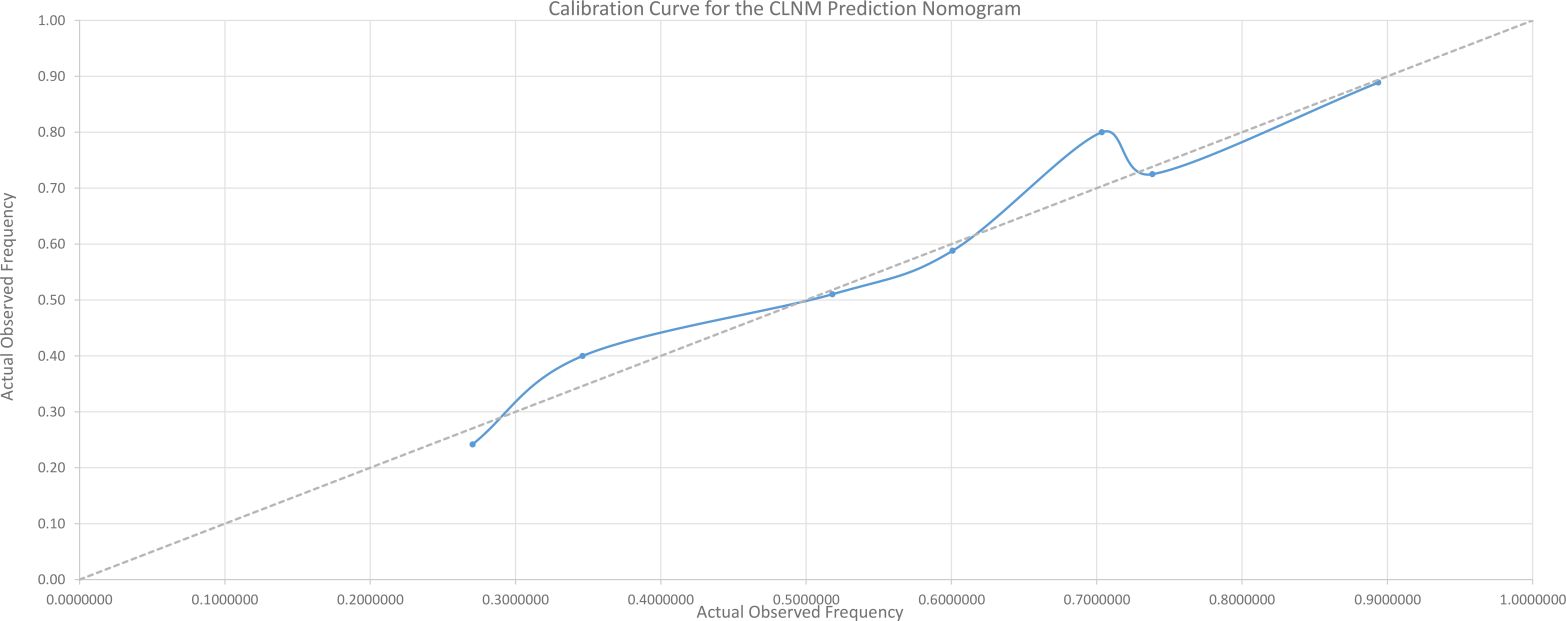
Calibration curve for the CLNM prediction nomogram assessed by bootstrap resampling (n=1000 repetitions). The solid line represents the agreement between the nomogram-predicted probability and the actual observed frequency of CLNM after bootstrap correction. The dashed line represents the ideal reference. Close alignment indicates satisfactory model calibration.

We performed rigorous internal validation to evaluate the nomogram’s predictive performance. The model demonstrated acceptable discriminative ability, with a concordance index (C-index) of 0.712 (95% CI: 0.668–0.755),which is equivalent to the area under the receiver operating characteristic curve (AUC). The 95% confidence interval was calculated using the DeLong method. Internal validation using bootstrap resampling with 1000 repetitions further confirmed its robustness and calibration. The bootstrap-corrected calibration curve (**Figure 2**) showed good agreement between the nomogram-predicted probabilities and the actual observed outcomes, indicating satisfactory calibration of the model validated through the bootstrap resampling procedure.

### 3.4 Stratification power of high-risk co-mutation in microPTCs

Given that 55.7% (290/521) of the cohort consisted of microPTCs (tumor size ≤1 cm), and considering the evolving clinical paradigm towards active surveillance for such tumors, we specifically evaluated the predictive value of the high-risk multi-gene co-mutation within this subgroup.

Among the 290 patients with microPTCs, 11 (3.8%) harbored the high-risk co-mutation (BRAF/TERT/PIK3CA/TP53). The CLNM rate in these high-risk microPTCs was 81.8% (9/11), which was significantly higher than the rate of 46.2% (129/279) observed in microPTCs without the co-mutation. This difference corresponded to a >5-fold increase in the odds of CLNM (Odds Ratio [OR] = 5.23, 95% Confidence Interval [CI]: 1.21 - 22.66; P = 0.027, Fisher’s exact test).

This subgroup analysis confirms that the high-risk co-mutation serves as a powerful discriminator of biological aggressiveness even within microPTCs, highlighting its potential clinical utility for risk stratification in the context of active surveillance decision-making.

### 3.5 Risk factors for multiple lymph node metastases (>5 nodes) in central and lateral cervical regions

#### 3.5.1 Univariate analysis of risk factors for multiple lymph node metastases (>5 nodes)

LNM occurred in 60.46% (315/521) of patients, of which 71.43% (225/315) had 1–5 nodes involved, and 28.57% (90/315) had >5 nodes involved. The rate of multiple lymph node metastases (>5 nodes) was significantly higher in patients with tumor size >1 cm, bilateral lesions, tall cell/columnar cell/sclerosing subtypes, extrathyroidal extension, and lymphovascular invasion (**Table 3**).

#### 3.5.2 Multivariate analysis of risk factors for multiple lymph node metastases (>5 nodes)

Variables with P < 0.05 in the univariate analysis were included in the multivariate logistic regression model. Independent risk factors for multiple lymph node metastases (>5 nodes) were: tumor size >1 cm, bilateral lesions, and gross extrathyroidal extension (**Table 7**), consistent with previous studies (7).

**Table 7:** Multivariate analysis of multiple LNM (>5 nodes) in central and lateral cervical regions.

Variable	B	SE	Wals	P value	OR	95%CI
Size (>1 cm)	1.432	0.315	20.623	<0.001	3.908	2.257-7.772
Location (Bilateral)	0.658	0.188	12.293	<0.001	1.931	1.337-2.790
Extrathyroidal extension						
gross vs. none	2.232	0.795	7.885	0.005	9.315	1.962-44.223

## 4 Discussion

### 4.1 Summary of principal findings

This study establishes a comprehensive risk model for preoperative prediction of lymph node metastasis (LNM) in papillary thyroid carcinoma (PTC) by integrating next-generation sequencing (NGS) data with detailed clinicopathological characteristics. Our analysis unequivocally identifies high-risk multi-gene co-mutations (*BRAF* with *TERT*, *PIK3CA*, or *TP53*) —as the most powerful independent predictor for central LNM (CLNM) (OR = 6.319, 95% CI: 1.738–22.976, P = 0.005). Furthermore, male sex, age <45 years, bilateral lesions, extrathyroidal extension (ETE), and lymphovascular invasion (LVI) were also established as significant risk factors for CLNM and/or lateral LNM (LLNM).

### 4.2 The central role and mechanistic insights of multi-gene co-mutation

A pivotal finding of this study is the superior prognostic value of multi-gene co-mutations over the solitary *BRAF* V600E mutation in predicting aggressive behavior in PTC. This aligns with evolving perspectives in the field. For instance, Vuong et al. demonstrated that *BRAF* co-mutation with *TERT* promoter confers worse prognosis than single mutations (14). Similarly, a 2024 meta-analysis by Li et al. strongly supports our observation regarding the aggressive potential of *BRAF/TP53* co-mutations, confirming *TP53* as a robust marker of poor differentiation in *BRAF*-mutant PTC (17). The concept that complex mutational profiles signify advanced disease is further echoed by Yang et al., who linked multi-gene synergy to extensive lymph node metastasis (18).

The mechanistic basis for this strong association, particularly with CLNM, lies in the synergistic interaction of disrupted key pathways:

- *BRAF* V600E drives constitutive MAPK pathway activation, promoting proliferation and survival (8, 9).
- *TERT* promoter mutations maintain telomere length, conferring cellular immortality essential for metastatic clone persistence (9, 12).
- *PIK3CA* mutations activate the *PI3K-AKT* pathway, which cooperates with *MAPK* signaling to enhance invasive behavior and adaptation (19).
- *TP53* inactivation impairs apoptosis, allowing cells with proliferative advantages to evade cell death (15, 17).

When co-occurring, these alterations establish a multi-pathway network that collectively potentiates metastatic competence. The cross-talk between *MAPK* and *PI3K-AKT* pathways, coupled with *TERT*-mediated telomere maintenance and *TP53*-related apoptotic escape, creates a molecular context highly permissive for lymph node invasion and colonization (17, 18, 20). This integrative biology explains why *BRAF* co-mutated with *TERT*, *PIK3CA*, and/or *TP53* emerged as the strongest independent predictor of CLNM in our cohort.

Notably, our study also identified a subset of tumors (5.6%, 29/521) lacking any detectable driver mutation within our panel. This finding underscores the molecular heterogeneity of PTC. The wild-type status in these cases may be attributed to several factors, including: 1) oncogenic gene fusions (e.g., involving *RET*, *NTRK1/2/3*, or *ALK*) that are not optimally detected by DNA-based panels and require RNA sequencing; 2) non-coding alterations such as *TERT* promoter mutations or enhancer rearrangements; 3) activation of alternative signaling pathways (e.g., Wnt/β-catenin, Hippo) through mutations in genes not covered by our panel (e.g., *AXIN1*, *APC*, *NF2*); or 4) technical/biological factors like low tumor cellularity. This highlights the importance of employing broader molecular profiling strategies (e.g., RNA sequencing, whole-exome sequencing) in future studies to fully characterize the genomic landscape of PTC, especially in mutation-negative cases.

Therefore, our study advances the field by systematically validating this high-risk molecular constellation in a large Chinese cohort and translating this knowledge into a clinically applicable, quantitative nomogram that integrates molecular signature with clinicopathological features for preoperative risk assessment. Notably, we demonstrate its potent discriminatory power even within the microPTC subgroup, addressing a critical dilemma in conservative management. The importance of such multi-gene profiling for risk stratification is increasingly recognized, as reflected in both international guidelines (11) and domestic expert consensuses (21). Recent studies leveraging comprehensive molecular profiling, such as that by Yoo et al. (2022), have further highlighted the potential of integrating multi-omics data for refined risk assessment (22).

### 4.3 Comparison with previous studies and consideration of potential discrepancies

To contextualize our findings, it is pertinent to systematically compare them with key previous studies and to discuss potential sources of divergence. Our results strongly align with the growing consensus that multi-gene co-mutations, particularly involving *BRAF*, *TERT*, *PIK3CA*, and *TP53*, denote a highly aggressive subset of PTC. The prognostic synergy of *BRAF* and *TERT* promoter mutations reported by Vuong et al. (14), and the association of *TP53* mutation with adverse outcomes in *BRAF*-mutant PTC confirmed by Li et al. (17), are directly corroborated by our data. Similarly, the link between complex mutational profiles and extensive lymph node metastasis observed by Yang et al. (18) is consistent with our findings, reinforcing the translational relevance of these molecular signatures.

Notably, the prevalence and specific predictive values of these co-mutations in our cohort may exhibit certain variations when compared to some reports from Western populations, such as the TCGA cohort (23), although recent comparative studies highlight ethnic variations (24). Several non-mutually exclusive factors may contribute to these differences. First, ethnic and racial factors may influence the underlying genetic landscape of PTC. Our study comprised exclusively Chinese patients, and population-specific genetic backgrounds or environmental exposures could modulate mutation frequencies and phenotypic expression. Second, differences in sample selection criteria must be considered. Our study enrolled consecutive treatment-naïve patients, which may reflect a broader clinical spectrum compared to cohorts enriched for advanced or recurrent disease. This difference in baseline risk could affect the observed association strengths between molecular markers and outcomes like LNM. Third, technical discrepancies in molecular profiling exist. While we utilized a targeted 88-gene NGS panel with high depth (>500x), differences in sequencing platforms, gene coverage, variant-calling algorithms, and sensitivity thresholds across studies can lead to variances in mutation detection rates, particularly for low-frequency alterations.

Despite these potential sources of variation, the internal consistency of our findings and their alignment with major mechanistic principles support their validity. Our study contributes a well-characterized Chinese cohort to the global literature and underscores the universal importance of multi-gene synergy, while also highlighting the need for population-specific validation of molecular risk stratification tools before broad clinical implementation.

### 4.4 Complementary value of clinicopathological factors and clinical implications

Despite the powerful predictive strength of molecular markers, traditional clinicopathological factors remain indispensable for risk assessment and complement molecular indicators. This study confirms that gross ETE is one of the most significant risk factors for LLNM and multiple LNM (>5 nodes) (OR>21), consistent with previous studies (25–27), highlighting the critical role of local invasive capacity in metastasis. Additionally, tumor size >1 cm and bilateral lesions were also identified as independent predictors for LLNM and multiple LNM (7, 26).

These findings indicate that a comprehensive preoperative risk assessment system should integrate molecular signatures (e.g., high-risk multi-gene profiles) and anatomical/clinical features (e.g., tumor extent, ETE). To operationalize this integrated approach, we developed a nomogram (**Figure 1**) that provides clinicians with a practical tool to individualize surgical planning. This model facilitates more precise identification of high-risk patients who are likely to benefit from prophylactic central and even lateral compartment lymph node dissection.

**It i**s important to delineate the clinical scope of our findings. This study focuses on molecular risk stratification to guide surgical decision-making, not on recommendations for systemic therapy. BRAF/MEK inhibitors, for instance, are currently indicated only for advanced, radioiodine-refractory disease, a context distinct from that of our treatment-naïve surgical cohort. However, the comprehensive molecular profile we establish provides a foundational biological map of the tumor. This information, in conjunction with clinical stage, could inform long-term surveillance strategies and become relevant for future therapeutic considerations should disease progress.

The development of our preoperative nomogram aligns with the current trend towards personalized surgical planning, as seen in recent efforts by Mu et al. (28) and Hwang et al. (29). Moreover, emerging models that incorporate other molecular signatures alongside mutations, as demonstrated by Wang et al. in their nomogram for predicting lateral lymph node metastasis (30), represent a promising direction in precision risk assessment.

However, our model distinguishes itself by unequivocally identifying and quantifying the dominant predictive power of high-risk multi-gene co-mutations, which emerged as the strongest independent risk factor for CLNM. This provides a more profound molecular biological basis for risk stratification compared to models that rely primarily on clinical and ultrasonographic features. Furthermore, the validation of our model’s utility in the microPTC subset offers a direct and novel tool for refining decision-making between active surveillance and surgery. In addition to affirming the value of clinicopathological factors, our integrated modeling approach also allowed for a detailed dissection of their nuanced and context-dependent roles. For instance, tumor size >1 cm was a strong independent predictor for lateral and multiple lymph node metastases, but not for central compartment metastasis in the multivariate model. This suggests that its influence on central nodal spread may be indirect and mediated through more proximate biological features such as lymphovascular invasion or specific molecular co-alterations. Conversely, factors like younger age (<45 years) remained independent predictors across models, though their effect sizes (e.g., OR = 1.75 for LLNM) were quantitatively less dominant when compared to powerful anatomical risk factors like gross extrathyroidal extension (OR = 21.99).These findings underscore the complexity of metastatic patterns and the value of integrated models that weigh both clinical and molecular determinants.

### 4.5 Translation into clinical practice

The potent predictive value of high-risk co-mutations holds particular clinical promise for refining the management of microPTCs (≤1 cm). Our sub-analysis revealed that within microPTCs (n=290), those harboring high-risk co-mutations had an 81.8% rate of central lymph node metastasis (CLNM)—a more than fivefold increase in risk compared to those without (OR = 5.23, P = 0.027). In the contemporary era where active surveillance is an option for microPTCs (4), this molecular signature provides a powerful tool to identify the small but significant subset of tumors with inherently aggressive potential.

Therefore, integrating our model into clinical practice enables a more personalized, molecularly informed surgical strategy. We propose the following clinical pathway: perform NGS on preoperative FNA samples. If a high-risk multi-gene profile is identified, it provides a compelling rationale for undertaking a comprehensive central compartment dissection, even with equivocal imaging findings for central nodes.

Crucially, this molecular signal pertains primarily to the central compartment. It does not justify prophylactic lateral neck dissection (LND) in the absence of imaging evidence. Lateral neck management must remain strictly dictated by preoperative ultrasound (± CT) and intraoperative assessment. The value of our model lies in heightening suspicion for biologically aggressive disease, prompting meticulous central compartment surgery and surveillance, thereby potentially reducing reoperation rates while avoiding unwarranted lateral neck morbidity.

This approach is especially pivotal for microPTCs, where it can directly inform the critical choice between initial active surveillance and upfront therapeutic surgery.

It is also important to acknowledge the limitations of our study. As a retrospective investigation based on archival tissue, we lacked systematic data on potential environmental risk factors (e.g., radiation exposure, tobacco use) and lifestyle factors (e.g., diet, body mass index) for the cohort. While this does not affect the validity of the identified molecular-clinical associations, such information could provide valuable additional context for the observed molecular patterns and represents an important direction for future prospective studies.

### 4.6 Study limitations and future perspectives

Our study has several limitations. First, as noted, patient management followed the 2012 ATA guidelines, reflecting a more aggressive surgical era. This may affect the generalizability of our absolute risk estimates to current practice. However, we posit that the very identification of high-risk co-mutations as the strongest predictor within this context underscores their profound biological significance, suggesting they remain critical discriminators in today’s more conservative paradigm.

Second, the statistical power of our study is limited by the sample size, particularly for the analysis of rare, high-order multi-gene interactions. Only 20 patients (3.8%) harbored the predefined high-risk co-mutation (*BRAF* with *TERT*, *PIK3CA*, and/or *TP53*). While bootstrap internal validation supports the robustness of our model’s overall performance, risk estimates for such specific, small subgroups should be interpreted with caution and require validation in larger cohorts.

Third, while internally validated via bootstrap resampling, our model lacks external validation in an independent cohort, which is critical for confirming its broad applicability.

Fourth, the study lacks clinical follow-up data. Consequently, we cannot analyze associations with long-term outcomes such as recurrence or survival. It is acknowledged, however, that the primary aim of our model was preoperative risk prediction for surgical planning; this limitation does not diminish its utility for that intended purpose. Establishing the full prognostic value of these co-mutations remains a crucial goal for future research.

Looking forward, large-scale, multicenter, prospective studies with long-term follow-up are essential to externally validate this risk stratification strategy. Future work should also integrate NGS-based molecular subtyping with high-resolution imaging and artificial intelligence to develop more powerful, visualizable preoperative prediction tools. The ultimate goal is to advance PTC management towards a paradigm that optimizes oncological control while minimizing unnecessary morbidity.

## Data Availability

The dataset generated for this study has been deposited in the Dryad repository and is publicly available at: https://doi.org/10.5061/dryad.bvq83bkpx.
