# Correction: Multi-gene co-mutations of BRAF with TERT, PIK3CA, or TP53 are powerful predictors of central lymph node metastasis in papillary thyroid carcinoma

**DOI:** 10.3389/fendo.2026.1798213

**Published:** 2026-02-27

**Authors:** Qing Yu, Han Liu

**Affiliations:** Department of Pathology, Deyang People’s Hospital, Deyang, China

**Keywords:** lymph node metastasis, multi-gene co-mutation, next-generation sequencing (NGS), papillary thyroid carcinoma, risk factors

The **Data Availability Statement** was erroneously given as “The original contributions presented in the study are included in the article/Supplementary Material”.

The correct **Data Availability Statement** is “The dataset generated for this study has been deposited in the Dryad repository and is publicly available at: https://doi.org/10.5061/dryad.bvq83bkpx“.

The original version of this article has been updated.

