# Correction: Multi-gene co-mutations of BRAF with TERT, PIK3CA, or TP53 are powerful predictors of central lymph node metastasis in papillary thyroid carcinoma

**DOI:** 10.3389/fendo.2026.1925037

**Published:** 2026-07-22

**Authors:** Qing Yu, Han Liu

**Affiliations:** Department of Pathology, Deyang People’s Hospital, Deyang, China

**Keywords:** lymph node metastasis, multi-gene co-mutation, next-generation sequencing (NGS), papillary thyroid carcinoma, risk factors

In the published article, there was an error in the reference list.

Due to a systematic error during the export from the reference management software, the majority of the references were incorrectly cited. The reference list has been fully corrected and is provided below in its entirety.
